# Role of Winter Weather Conditions and Slipperiness on Tourists’ Accidents in Finland

**DOI:** 10.3390/ijerph13080822

**Published:** 2016-08-15

**Authors:** Élise Lépy, Sinikka Rantala, Antti Huusko, Pentti Nieminen, Marjo Hippi, Arja Rautio

**Affiliations:** 1 Faculty of Humanities, University of Oulu, 90014 Oulu, Finland; 2 Faculty of Medicine, Arctic Health, University of Oulu, 90014 Oulu, Finland; arja.rautio@oulu.fi; 3 Medical Research Center Oulu, Oulu University Hospital, 90220 Oulu, Finland; sinikka.rantala@ppshp.fi; 4 Thule Institute, University of Oulu, 90014 Oulu, Finland; antti.huusko@oulu.fi; 5 Medical Informatics and Statistics Group, University of Oulu, 90220 Oulu, Finland; pentti.nieminen@oulu.fi; 6 Finnish Meteorological Institute, 00560 Helsinki, Finland; marjo.hippi@fmi.fi

**Keywords:** climate, slipping hazards, tourist patients, extremity injuries, weather

## Abstract

(1) Background: In Finland, slippery snowy or icy ground surface conditions can be quite hazardous to human health during wintertime. We focused on the impacts of the variability in weather conditions on tourists’ health via documented accidents during the winter season in the Sotkamo area. We attempted to estimate the slipping hazard in a specific context of space and time focusing on the weather and other possible parameters, responsible for fluctuations in the numbers of injuries/accidents; (2) Methods: We used statistical distributions with graphical illustrations to examine the distribution of visits to Kainuu Hospital by non-local patients and their characteristics/causes; graphs to illustrate the distribution of the different characteristics of weather conditions; questionnaires and interviews conducted among health care and safety personnel in Sotkamo and Kuusamo; (3) Results: There was a clear seasonal distribution in the numbers and types of extremity injuries of non-local patients. While the risk of slipping is emphasized, other factors leading to injuries are evaluated; and (4) Conclusions: The study highlighted the clear role of wintery weather conditions as a cause of extremity injuries even though other aspects must also be considered. Future scenarios, challenges and adaptive strategies are also discussed from the viewpoint of climate change.

## 1. Introduction

### 1.1. Health and Well-Being in the North

It is now accepted that the ambient conditions of Arctic and sub-Arctic communities are affected by the variability of climate and environmental changes [1], and these exert a direct impact on health, subjective well-being and quality of life [2]. While many investigators have assessed the impact of global warming on the health of indigenous people [2,3,4] most have focused on food and water security, infectious diseases, increased risk of contaminants and health care infrastructure [2,5,6]. On contrast, the present study considers the impacts of the variability in the weather conditions during the winter on the health and safety of people undertaking outdoor activities in Northern Finland. Indeed, pedestrians, bicyclists and skiers may fall and hurt themselves due to the changes in the ground surface conditions attributable to the vagaries in the weather.

In Finland, snowy or icy ground surface conditions can be rather hazardous to human health during wintertime. Studies in ergonomics have shown that slip-and-fall accidents occur more often during the winter months [7,8,9] and it is not only outdoor workers but also the general public that suffer an elevated risk of slipping and falling [8]. Berggård [9] has estimated that “more than 100,000 pedestrians in the Nordic countries are every winter expected to receive medical treatment due to winter weather and slippery conditions”. According to several reports [10,11,12], there are between 50,000 and 70,000 pedestrian and cyclist accidental falls every year in Finland. Elderly people have been the focus of many of these investigations [13,14,15,16]; this reflects their vulnerability to the risk of distal radius fractures [17]. Some studies have also dealt with injuries related to winter sports, especially concentrating on accidents involving children and adolescents on the ski slope [18,19]. For example, Bouter et al. [20] have noted that the accident risk is elevated when there are patches of ice on the slopes used for downhill skiing and snowboarding causing injuries to the knees, ankles [21], wrists, thumbs and shoulders [22,23].

### 1.2. The Risk of Injury

The concept of risk is commonly described by the product of the vulnerability and the hazards but neither the hazard nor the vulnerability is easily measured [24]. In this research context, slippery conditions are regarded as hazards [25] if we define the hazard as “a condition with the potential of causing an adverse effect, for example, injury to people (…)” [26] and as limited in time and space with a high intensity. We have used the definition of Grönqvist et al. [25] who defined slipperiness as “conditions underfoot which may interfere with human beings, causing a foot slide that may result in injury or harmful loading of body tissues due to a sudden release of energy”. The concept of vulnerability has been defined in many research fields [27], such as environmental investigations [28,29,30,31] especially climate-related studies [32,33,34]. When assessing the vulnerability of people to slippery conditions, we need to consider several mutually interacting factors: the actual exposure of the individual, his/her perception of danger and the sensitivity to the hazard. In health related research, the definition of exposure slightly differs from that used in geographical studies, where the concept of risk is central. It might then be defined as “the presence of a substance (factor) in the environment external to the (person)” [35]; in this study, that substance is water, in all its many forms, lying on the surface of the ground. 

### 1.3. Slippery Conditions

According to Grönqvist et al. [25], injuries caused by slips are not the result of trivial incidents but of a multitude and complexity of risk factors. Subsequently, Gao and Abeysekera [36] devised a comprehensive systems model of factors contributing to slip-and-fall accidents on icy and snowy surfaces in Sweden. This model, which can be applied for all Nordic regions, includes extrinsic (environmental), intrinsic (human) and mixed (footwear/surface interaction, etc.) risk factors. Norlander et al. [7] have also divided the perceived risk factors into four categories: the physical environment (slippery and sloping surfaces, low visibility and inaccessibility), risky work situations, individual factors (safety behavior, human gait, etc.) and organizational factors (street maintenance, equipment, etc.). In this study, we will focus on the environmental factors and underfoot surface characteristics.

In fact, winter environmental conditions are extremely present in Arctic and sub-Arctic regions that factors such as temperature, snowfall and darkness can greatly affect how slippery and dangerous pavements can be [8]. It is recognized that slips and falls are often attributed to slippery surfaces, in other words they have low friction properties [7]. In wintertime, there are various types of underfoot surfaces such as ice, snow, melting ice or/snow, compressed snow, ice covered with snow [37] and thus the degree of slipperiness will differ depending on the surface temperature, structure, hardness and thickness of the water layer [8].

### 1.4. Aim

In order to formulate the objectives of this study, some prerequisites need to be specified. First of all, tourism is a major part of the local economy and good tourist resorts have to provide not only accommodation and outdoor facilities but also to ensure safety and healthcare [38]. Second, winter-sports and other outdoor activities in Finland are climate-dependent and therefore vulnerable to meteorological variations [38,39]. The main objective of this study was to focus on the impacts of the variability of weather conditions on the health of tourists assessed via documented accidents occurring during the winter season in the Sotkamo tourist area. We attempted to estimate the slipping hazard exposures in a specific context of space (tourist centre) and time (winter season) and we have focused not only on the weather but also other relevant parameters (e.g., holidays, seasonal variations) which could be responsible for any increases in the numbers of injuries and accidents. This is the first study to investigate all-aged tourists’ health and to combine weather data with different types of injuries. 

This study aims to answer to the following research questions: How are tourists affected by wintery weather conditions? What other factors play a role in the number and type of slip-and-fall injuries (unfamiliarity with icy and snowy environments, changes in behavior when on holiday, etc.)? What are the relationships between tourists, climate change and health care?

## 2. Materials and Methods

Located in Northern Finland, Sotkamo is one of the most popular Finnish all-year-round tourist destinations although the majority (62%) of the tourists visit during the winter season. The municipality belongs to the second highest tourism municipality category defined in a national review of tourism [40]. In 2009, Sotkamo recorded over 600,000 overnight stays in registered accommodation with 11% of these by foreign visitors, mainly from Russia [41]. Since it is surrounded by lakes, rivers and hills, the Sotkamo region, and especially the Vuokatti resort centre, can offer a wide range of activities throughout the year: cross-country skiing, downhill skiing, snowmobiling, safaris and other ice-related activities in the winter; boating, fishing, hunting and hiking in the summer. While domestic and international tourism growth has been increasing in the Kainuu region, in recent years tour operators and local tourism entrepreneurs have had to cope with weather fluctuations. Indeed, the variability of extreme temperatures and different types of precipitation, with fewer snow days, less snow depth and the uncertain freezing conditions can have a negative impact on a resort’s image leading to increased vulnerability of the regional tourism sector [42]. It is clear, the climate has an important role in drawing a tourist destination image [43] as the landscape perception of tourist [44] might be decisive in determining a destination.

In order to meet the objective of the present study, a multidisciplinary investigation has been conducted combining quantitative and qualitative data treatment and analysis.

### 2.1. The Study Population 

In the tourist resort of Vuokatti, medical assistance can be soughtfrom the health centre in the municipality of Sotkamo which is situated 7 kilometres south-east from the ski resort. Sotkamo Health Centre is under the auspices of the Joint Authority of Kainuu Region; it provides basic medical care for minor injuries and other acute health problems. It is the primary contact place for a tourist who is injured and/or ill. In more severe cases and for more specialized medical needs, patients are sent from the Sotkamo health centre to the Kainuu Hospital which is located in Kajaani 40 kilometres west of Sotkamo. Outside of opening hours of the Sotkamo health centre (8 a.m.–6 p.m. on weekdays, 9 a.m.–6 p.m. at weekends), Kainuu Hospital emergency service provides medical care for all patients who cannot wait until the next day.

The study population consisted of injured tourists. We received ethical permission to access the data of non-local health service users from Kainuu Hospital. This study focuses on the patients who were injured in the following parts of the body: shoulder and upper arm (S40–S49 according to the ICD-10 diagnosis codes), elbow and forearm (S50–S59), wrist and hand (S60–S69), hip and thigh (S70–S79), knee and lower leg (S80–S89) and ankle and foot (S90–S99). We assume that the above-mentioned extremity injuries most commonly occur after slips and falls. The data was extracted for three winter seasons from 15 October to 15 April of 2006/2007, 2007/2008 and 2008/2009. 

Kainuu Hospital has data on the year of birth, the age, the gender, the home residence, the home country, the date of the visit and the ICD-10 diagnosis codes. During the three studied winter seasons, a total of 1596 patients had visited the hospital with 336 of them being diagnosed with the above-mentioned injuries (S40–S99).

### 2.2. Questionnaires and Interviews of Health and Safety Personnel

Questionnaire and interviews were used in the framework of the VACCIA project for determining the attitudes of health and security personnel to climate change and for obtaining more information on risks of injuries, risk categories of patients and so forth. Although this study specifically focuses on the Sotkamo area, these qualitative methods were applied for both Sotkamo and Kuusamo. Since Kuusamo is a comparable case in terms of tourism [38], we decided to use the results from both locations.

The questionnaire was distributed to all staff working in the emergency service of the health care centres in Sotkamo and Kuusamo (altogether seven answers). It included three questions about the respondents’ backgrounds and the following four series of questions: *(1) What is the impact of travellers in the overall health centre’s operations (in term of emergency rooms, injuries, communication etc.)? In what ways working with travellers is different than working with locals? Describe the typical traveller/patient; (2) Have there been any changes in the percentage of travellers (as patients), the injury types or travellers’ age structure in recent years? If so, what kind of changes? (3) Have you noticed any connection between the weather and the type of patients coming to the health centre? (4) How do you see the future in Sotkamo/Kuusamo health care?*

Interviews were conducted with two individuals from the police and fire departments of Kuusamo and with the chief nurse in Sotkamo. The questions concerned the interviewee’s own organization and their future strategies towards climate change. 

### 2.3. Workshops

Within the context of the VACCIA project, participatory work took place in Sotkamo and Kuusamo and included two workshops organised in both municipalities in 2009 and 2010 where researchers and stakeholders such as local tourism entrepreneurs, state officials, tourism developers and representative of social, healthcare and security sectors of the respective municipalities discussed these issues. The main topic was how could tourism in Northern Finland best adapt to the climate change [38]. Healthcare related issues were examined as part of these discussions.

### 2.4. Weather-Related Data and Slippery Conditions

Weather-related data was provided by the Finnish Meteorological Institute (FMI) for the three time periods. It includes the following parameters at hourly intervals: time (year, month, day and hour), air temperature (TAmb in °C), dew point temperature (TDew in °C), surface temperature (Tmp in °C), relative humidity (Rhz in %), precipitations (Prec in mm/h), surface cover as water (SrfWat in mm), snow (SrfSnow in mm) and ice (SrfIce in mm). The information about surface temperature and surface cover has been estimated by the FMI’s road weather model [45,46] which is a physical energy balance model providing information on the conditions on and below the road surface due to weather. The model makes an estimate about surface cover taking into account past and present weather. Surface cover is presented using storages (water, ice, snow) and storages can change (melting, freezing, evaporation, condensation, mechanical wear) or storages may also interact with each other, for example the size of the water storage increases due to precipitation as well as by melting of snow or ice. The unit of measurement is given in mm and it represents the equivalent amount of water.

Based on the literature [12,47], six scenarios were devised with respect to slippery conditions. A classification was created of slippery conditions with their own characteristic weather variables leading to conditions for slipperiness (Table 1). This made it possible to determine which slippery scenarios occurred each day. Then, we linked the slippery conditions with each injury case. Since no information was available concerning the time when the patient was registered at the hospital nor on the time when the accident had actually occurred, a 36-h period was applied. For example, if a patient visited the emergency on 20 December, weather-related data from 19 December, 12 p.m. to 20 December, 11 p.m. was analyzed over the one-hour time frame. Slippery cases were selected if they occurred at least once during that 36-h period.

### 2.5. Data Analysis

Quantitative data (patients and weather) was analyzed using Microsoft Excel software (Microsoft, Redmond, WA, USA) and converted to graphics with Adobe Illustrator (Adobe Systems, San Jose, CA, USA). Statistical distributions with graphical illustrations were used to describe the distribution of non-local patients, their characteristics and their reasons for visits to Kainuu Hospital. Graphs were used to illustrate the distribution of the different characteristics of the weather conditions. As the total number of tourists was not available for this study, the injury rate could not be calculated.

Qualitative data (questionnaire and interview responses) was analyzed manually because of the small sample and the multiple-response questions.

## 3. Results

### 3.1. Seasonal Distribution of Injury Cases

All the responses of interviewees informed that there were seasonal variations in the emergency service visits. The summer and autumn times were less hectic, whereas the emergency services were much busier in wintertime since more people, especially tourists, were undertaking outdoor activities in cold and often slippery conditions. Local inhabitants tend to avoid going out during inclement weather. The interviewees stated that they associated slippery weather conditions with an increase in sprains, strains and fractures. They also noticed that during very cold winter days, there was a tendency that patients would not seek emergency early in the morning but wait until later during the day.

### 3.2. Non-Local Patients and Extremity Injuries

**Figure 1:**
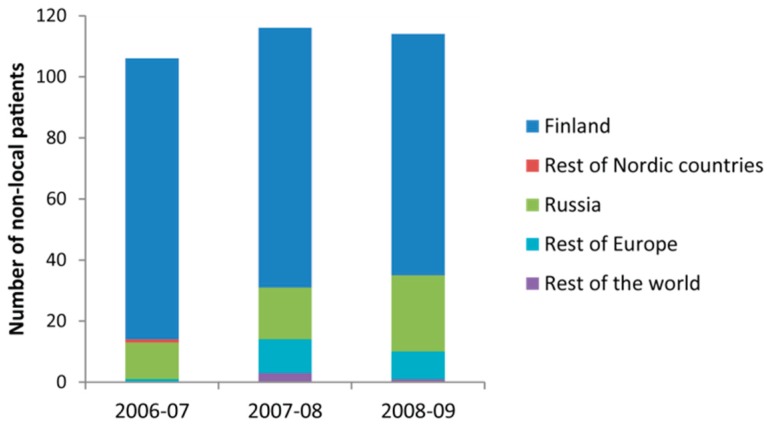
Origin of non-local of Kainuu Hospital patients whose injury belongs to S40–S99 category.

The patient database provided by Kainuu Hospital excluded locals, that is, inhabitants of Sotkamo or Kajaani municipalities. From this database, it can be seen that among the patients who suffered extremity injuries (S40–S99), 86.8% were Finnish travellers in 2006/2007, 73.3% in 2007/2008 and 69.3% in 2008/2009 (Table 2). During the three studied period, most of foreign patients were from Russia (Figure 1). Extremity injuries represented over 20% of all visits during the three studied period (Table 2).

The answers of the questionnaires revealed that the Russians were mentioned as the largest group of foreigners. Two other categories were also mentioned: children on school trips and Finnish tourists during their winter holidays. It also was estimated that travellers accounted for about one third of emergency patients in summer and even more in winter. 

**Figure 2:**
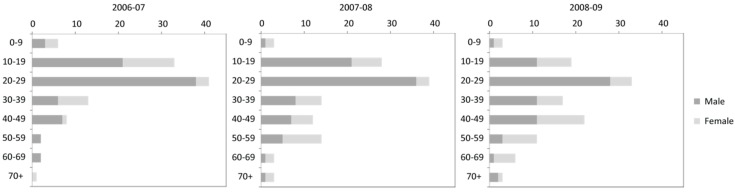
Number of injury cases by age and gender at Kainuu Hospital.

From the interviews and statistical data (Figure 2), the risk age category that was the most likely to experience an extremity injury were those in the 10–29 years old category, mostly boys and young men. With respect to elderly patients, it was more likely to be a woman and it was noted that there had been an increase in the numbers of visits to the emergency service by more elderly individuals for extremity injuries.

**Figure 3:**
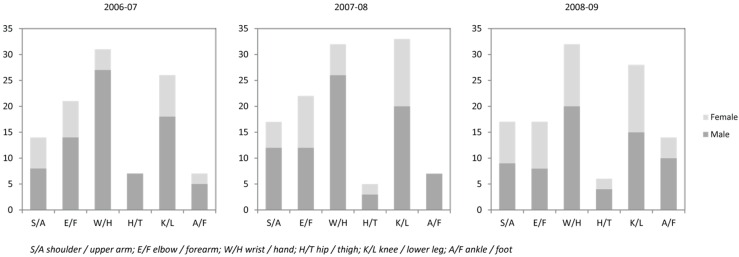
Number of injury cases by type and gender at Kainuu Hospital.

The time frame when fractures occurred was said to be widened because of the use of pipe (one type of snowboarding), but the actual numbers of fractures had not necessarily increased. Figure 3 shows that the typical injuries concerned mostly the wrist and hand and knee and lower leg.

### 3.3. Exposure to Slipping Hazards

**Figure 4:**
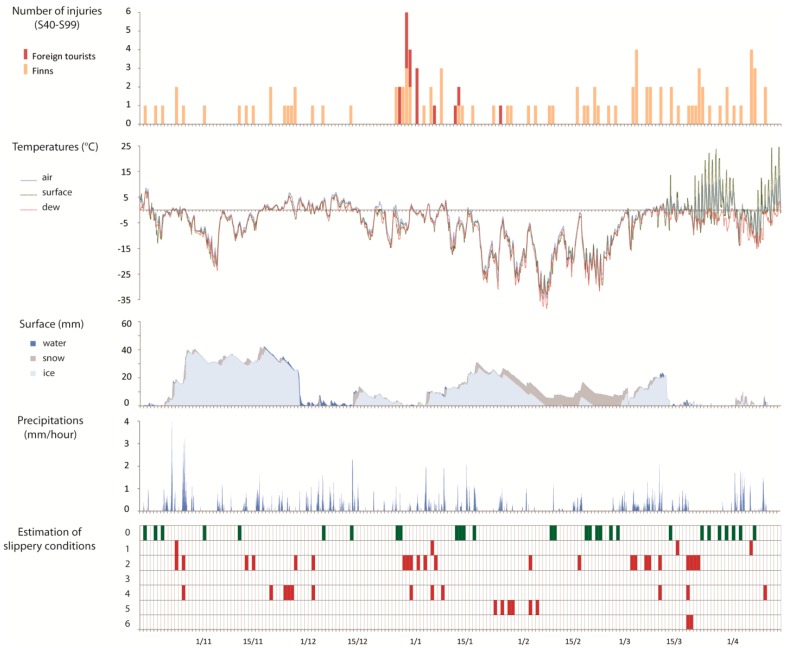
Injuries associated to weather conditions in 2006/2007.

**Figure 5:**
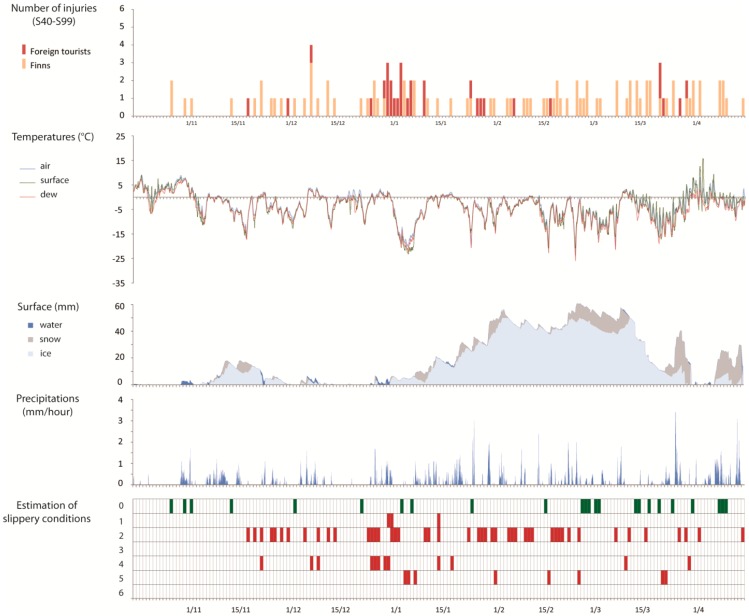
Injuries associated to weather conditions in 2007/2008.

**Figure 6:**
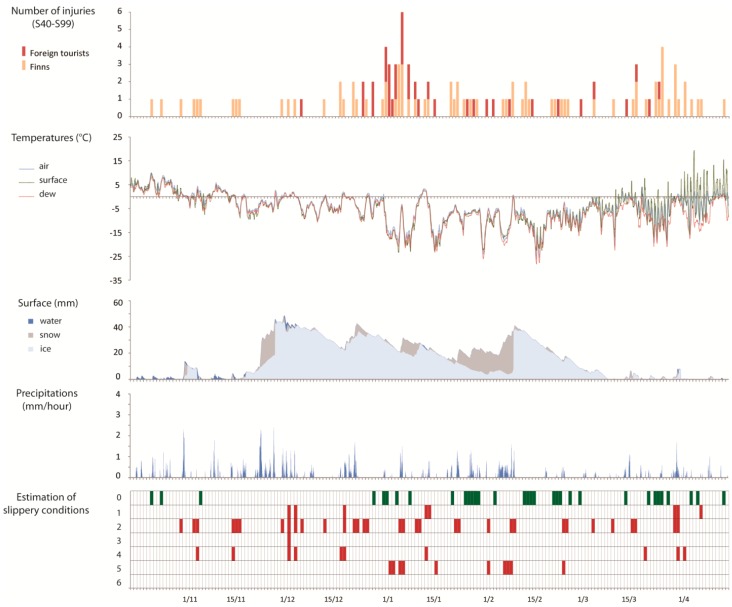
Injuries associated to weather conditions in 2008/2009.

The weather data analysis for the years 2006/2007, 2007/2008 and 2008/2009 was used to link the time when the patients were registered in Kainuu Hospital to the prevailing weather conditions (air and surface temperatures, dew point, surface cover and precipitations) which allowed us to make an estimation of slippery conditions in 36 h prior to the end of the day that the patients appeared for treatment. For each day that a patient was registered, slippery conditions that occurred at least once during this 36-h period were marked in the Figure 4, Figure 5 and Figure 6. The slippery scenarios are based on the conditions described in Table 1.

For the three seasons, it can be observed that about one out of every three and sometimes even more, of the injury accidents occurred when the ground was not at all slippery (Table 3). Slipperiness can therefore be discarded as the only reason for injuries. This situation appeared more at the end of the winter.

In 2007/2008, half of the injuries occurred when the water vapour was condensing and when the surface temperature was lower than the dew point (slippery case 2). In fact, this type of slipperiness was also the most common scenario in the other two study periods, accounting for over 40% of accidents in these years (2006/2007—43.4%; 2008/2009—43.9%). Scenarios 3 and 6, in other words precipitation of freezing fog and supercooled rain leading to the formation of black ice were rather rare during the study periods.

Overall, despite the small differences in percentage numbers, it can be observed that there was a certain degree of stability in the slippery conditions cases over the three seasons.

## 4. Discussion

Our results showed that winter weather conditions have an evident role in the numbers and types of injuries in a northern tourism resort but other characteristics have to be considered as well:

- The background characteristics of tourists are important. Some of them may be familiar with snowy and icy conditions but often these tourists are coming from places where they rarely, if ever, encounter snow and ice and they will be unfamiliar and unprepared to face the Nordic winter environment.
- Most of the responders mentioned that the number of tourists has increased significantly even though some of them also noted that the so-called slope culture had improved and therefore the emergency room is not as busy as before during the “Russian week” or skiing holiday weeks.
- According to the responses, the typical traveller/patient has usually hurt him/herself on a slope or has a flu, today’s travellers were also mentioned as being busier than before, and occasionally travellers were already sick when leaving home, which may contribute to the increasing number of injuries.
- There are now more elderly tourists and people with multiple health problems, in particular elderly women with osteoporosis may be at risk when surface conditions are slippery.
- Other factors such as alcohol consumption during special events like New Year’s Eve may increase the number of patients arriving at the emergency room.
- The quality of some municipal services such as keeping the roads clear of snow and ice has an important role.
- Adaptation to environmental changes will be essential in the future [38].

Moreover, responses to questionnaires and interviews support the belief that winter weather conditions cannot be considered as the only factor causing extremity injuries in this specific tourist resort. When evaluating future prospects with respect to climate change, we have to consider the following points:

1. The future climate predictions. The scenarios provided by the Finnish Meteorological Institute indicate that there will be more warming (in a range of 3–9 °C) and increased precipitations (by 10%–40%) in the winter than in summer (where temperatures are predicted to rise by 1–5 °C and precipitation to increase by 0%–20%) and these winter changes will have a greater impact in the north of the country [48]. These predictions suggest that there will be more freezing point days (with daily minimum temperature below zero and maximum temperature above zero) and therefore the number of frost and thaw cycles will increase in the future meaning that the occurrence of slippery conditions, especially rain on icy surface (scenario 4), will be more common.
2. The possible concentration of tourists during certain periods due to the possible shortening of winter season in the future. This will directly affect the management of health care services.
3. The ageing of the population. The respondents expect that there will be an increase in the number of older and more frail tourist patients who will need health care during their holiday trip. On the other hand, the fluctuations in wintery weather conditions may deter older people from moving outside [49].

Findings of this study have been discussed with local policy-makers and service producers for evaluating current and future adaptation strategies for health care services and safety of tourists in Sotkamo. Tourism entrepreneurs have stated there that they would be willing to take into account and participate to ensure the well-being of tourists in the future. Health care and security representatives emphasized that their services might be affected by the changing weather conditions, for instance more freezing and thaw cycles would be anticipated to increase the numbers not only of falls and slips but also traffic accidents, especially with drivers inexperienced with winter conditions. As Berggård [9] stated, measures to reduce the risk of accident can be either community-based or depend on individual initiatives. In our case study, even though technical remedies such as the use of sand and salt have been utilized for pavement and road safety, health and security representatives emphasized that accurate information should be targeted to tourists as part of future adaptation strategies to prevent road accidents [42]. The Finnish Meteorological Institute has recently started to forecast local weather and warnings of winter sidewalk and street maintenance. Gao and Abeysekera [36] devised a term they called systemic prevention by which they meant aspects like the provision of walk aids and training especially for older people and the improvement of lighting conditions especially at high latitudes where the darkness prevails for most of the day in autumn and winter. Moreover, pedestrians should be advised to use appropriate anti-slip footwear to prevent falls and slips on snowy and icy surfaces [37]. In addition, reinforcing the culture of safe and friendly skiing among the adolescents and young adults would decrease the risk behavior leading to accidents.

Future adaptation strategies will directly impact on the management of patients and health care services. In the discussion about future prospects, the majority of interviewees thought that the number of patients would remain about the same as present or increase. They speculated that future climate conditions, in other words a shortening of winter season and therefore more tourists visiting during an abridged period, could easily overwhelm current health care resources, the solution proposed was a reallocation of resources. For example, this would mean a decline of the availability of non-urgent health care for local inhabitants and an increased workload for the emergency services. It was also stated that it would be a challenge for health care services to provide acute medical care for the increasing number of ageing tourists. Overall, one of the most critical factors would be the economic health of the municipalities [42]. 

## 5. Conclusions

This study was undertaken to investigate the relationship between slipperiness and tourists’ accidents recorded in health care databases during three winter seasons in Finland. The quantitative analysis of weather data through the different slippery scenarios showed that winter weather conditions had a critical role in the tourists’ injuries. Nevertheless, tourists’ safety and health are also affected by other risk factors such as the number of tourists during holiday seasons, the status of tourists traveling (like infections, existing diseases), and the maintenance of roads and pavements. Relevant adaptation strategies and collaboration between tourism sector and health care system should be considered.

## Figures and Tables

**Table 1:** Determination of slipperiness. (Finally, packed and compressed snow by walkers can also lead to the formation of harder and icy surface. Since this case is difficult to estimate, it has been discarded from this study).

Scenarios	Slippery Conditions	Weather Variables and the Conditions Causing Slipperiness
**0**	**Not slippery**	
**1**	**Freezing of water surface**	
	*This is mainly caused by a decrease of the surface and air temperatures before evaporation. It has the consequence of freezing the water on top of the surface. The ice layer is thin and transparent.*	TAmb < 0, Tmp < 0 and SrfWat > 0 (The quantity of water on the surface is taken at the same hour and one hour before)
**2**	**Solid condensation**	
	*This appears when the water vapour is condensing and when the surface temperature is lower than the dew point (surface temperature has to be below freezing). If the dew point is under 0 °C, there is no liquid phase but the condensation directly changes from water vapour into solid ice crystals. On the other hand, if the dew point temperature is above freezing, then there is a liquid phase of condensation and the water freezes when the temperature drops under 0 °C.*	Rhz > 95, Tmp < 0 and Tmp < Tdew (In this case, we consider that the amount of water vapour will be high enough if the relative humidity is greater than 95%)
**3**	**Precipitation of freezing fog**	
	*This can cause the formation of black ice in the autumn.* *It happens when the air temperature is below freezing and lower than the dew point and if the surface temperature is also below zero degrees.*	TAmb < 0, TAmb < TDew and Tmp < 0
**4**	**Rain on icy surface**	
	*“The surface will become very slippery when rain intensity is weak or the amount of precipitation is not very high. Heavy rain melts and makes the ice soft rapidly and no slippery surface can prevail”* [12].	0 < Prec < 4, SrfWat_+1_ > 0, SrfSnow = 0 or SrfSnow_+1_ < SrfSnow and SrfIce > 0 (Here, we consider light rain to be the case when precipitation is under 4 mm/h, as recommended by many meteorologists. Moreover, since the nature of the precipitation is unknown, we considered this to be the case when the surface is covered by water one hour later (+1) and when the snow cover is absent or decreasing)
**5**	**Snowfall on an icy surface**	
	*“It causes slipperiness when snow is dry enough. This occurs when air temperature is many degrees below zero. Snowfall can be very weak but still cause hazardous conditions. This case is actually worse than the (previous) case with water and ice. A snow covered surface may cause a surprise to pedestrians because it does not look slippery as an icy and wet surface does. When temperature increases above zero the snow starts to melt and friction” decreases* [12].	TAmb < −10, Prec > 0 and SrfIce > 0
**6**	**Supercooled rain, formation of black ice**	
	*This happens when rain is falling even although the air temperature is below freezing; black ice will be formed when the water comes into contact with a cold surface.*	Prec > 0, Tmp < 0, SrfWat = 0, SrfSnow = 0 and SrfIce_-1_ = 0 (In this case, we assume that the ground is dry, so the surface is not covered)

**Table 2:** Information on extremity injury cases treated in Kainuu Hospital.

Study Period	Number of S40–S99 Cases	Finns (%)	Foreigners (%)	Extremity Injuries/All Visits
October 2006–April 2007	106	86.79	13.21	22.13
October 2007–April 2008	116	73.25	26.75	21.13
October 2008–April 2009	114	69.30	30.70	20.07

**Table 3:** Slippery condition cases (%) for each injury case.

Slippery Condition Scenarios	2006/2007	2007/2008	2008/2009
0	35.8	32.8	42.1
1	8.5	5.2	9.6
2	43.4	50.0	43.9
3	0	0	0.9
4	19.8	17.2	10.5
5	5.7	9.5	16.7
6	1.9	0	0
